# Disparities of healthcare utilization in sexually transmitted diseases management: focusing on racial/regional variances with U.S. national inpatient sample in 2016–2019

**DOI:** 10.3389/fpubh.2025.1543117

**Published:** 2025-03-04

**Authors:** Jeong-Hui Park, Ji Eun Kim, Seohyun Woo, Sun Jung Kim

**Affiliations:** ^1^Department of Health Behavior, School of Public Health, Texas A&M University, College Station, TX, United States; ^2^Department of Medical Science, Soonchunhyang University Graduate School, Asan, Republic of Korea; ^3^Department of Health Administration and Management, College of Medical Science, Soonchunhyang University, Asan, Republic of Korea; ^4^Center for Healthcare Management Science, Soonchunhyang University, Asan, Republic of Korea

**Keywords:** health disparities, sexually transmitted diseases, NIS sample, healthcare utilization, racial and ethnic differences

## Abstract

**Objective:**

To identify patient and hospital factors, such as race and region, associated with increased sexually transmitted diseases (STDs) hospital charges, and emergency room (ER) usage for significant federal funding and research allocation.

**Methods:**

The National Inpatient Sample (NIS) of the United States was used to identify patients with STDs (weighted *n* = 22,275) from 2016 to 2019. The sample's characteristics, the odds of an ER visit, and the association between an ER visit and healthcare utilization measured by hospital charges were examined by multivariate logistic regression and linear regression.

**Results:**

Among 22,275 national inpatients, 74% had ER visits. The number of inpatients with STDs, ER visits, and average hospital charges continuously increased during the study period. Survey logistic results showed that sex, insurance type, and geographic region were associated with higher odds of ER visits among patients. The survey's linear results demonstrated that ER visits, Hispanic ethnicity, insurance type, and specific geographic regions were associated with higher hospital charges.

**Conclusions:**

Multiple factors are related to increased healthcare costs among patients with STDs, such as ER usage, Hispanic ethnicity, and insurance type. Policy efforts should focus on reducing ER dependency through targeted outreach, improving access to preventive care, and addressing disparities based on ethnicity and insurance status to reduce healthcare costs for vulnerable populations.

## 1 Introduction

Sexually transmitted diseases (STDs), also referred to as sexually transmitted infections (STIs), are a significant public health concern in the United States (US), encompassing various conditions such as chlamydia, gonorrhea, hepatitis, and HIV/AIDS (1, 2). These infections are transmitted through sexual activity and can progress to disease if left untreated, often remaining asymptomatic (2). In 2018, the US reported 67.6 million prevalent sexually transmitted infections cases and 26.2 million incidents, mainly in patients aged 15–24 years old (3). The asymptomatic nature of many STIs necessitates improved detection methods, such as point-of-care testing, which offer cost-effectiveness, timely diagnosis, and better patient follow-up (4). However, barriers to care, including confidentiality concerns and lack of access to healthcare, continue to impede efforts to combat STIs. In 2020, there were 1,579,885 cases of chlamydia, 677,769 cases of gonorrhea, and 133,945 cases of syphilis reported in the US (1), and these barriers persist, contributing to a steady rise in STDs rates (5).

Access to sexual health resources is essential for all individuals (particularly for those who seek care through STDs clinics), however, many are unaware of the services available (6). Also, many financial and social disparities surround STDs treatment at almost all levels; for example, individuals receiving HIV pre-exposure prophylaxis (PrEP) medications tend to be older, male, non-Hispanic, non-Black and possess commercial insurance (7). Moreover, a study found no significant difference in healthcare settings among patients with HIV, though women, those with higher incomes, and non-Hispanic Black individuals were more likely to visit hospital-based clinics (8, 9). Regional differences in healthcare costs also reflect disparities, with South Carolina showing higher emergency department costs for STDs and Maryland reporting higher outpatient and laboratory costs (10).

Geographically, the burden of STDs varies across the United States. Gonorrhea and syphilis cases have seen an increase in the Midwest, Northeast, and South, with higher rates observed among men and in Hispanic and Black communities (1, 11). The South, in particular, has notably high rates of HIV diagnosis, AIDS, and HIV-related mortality (1, 12, 13). Additionally, the Deep South exhibits the lowest rates of PrEP use and has historically received the least federal funding for HIV care and prevention (12). Rural areas face similar challenges, as the rural South experiences some of the highest rates of new HIV diagnoses, exacerbated by factors such as lack of Medicaid expansion, healthcare provider shortages, low health literacy, high STI rates, and stigma surrounding sexual health (1, 13, 14). For example, many primary care providers in these regions do not routinely screen for HIV, citing stigmas and resource constraints as significant barriers to care (15).

Despite STDs still remaining a critical public health issue in the US with significant disparities in healthcare access and utilization, the stigma surrounding sexual health, along with healthcare barriers, can hinder efforts to improve prevention and treatment. However, a gap remains in understanding the specific patient and hospital factors that contribute to the rising healthcare costs associated with STDs, particularly in terms of emergency room visits and hospital charges. Although studies have investigated disparities in STDs care, they have often focused on general healthcare utilization without fully exploring how socio-demographic factors, such as race, ethnicity, and region, intersect with healthcare costs in the context of emergency care. This study fills this gap by examining how race, geographic region, insurance type, and other factors uniquely contribute to increased emergency room visits and hospital charges for patients with STDs. The novelty of this study lies in its exploration of these associations on a national scale using a comprehensive dataset, offering a more granular understanding of how specific social and regional factors exacerbate healthcare burdens for STDs patients.

To address these disparities, further research is needed to examine differences in healthcare utilization across regions, racial groups, and healthcare costs. In this study, by identifying the factors contributing to disparities in hospital costs and emergency room visits, we can suggest resources and design interventions tailored to specific communities. The findings from this study will help inform efforts to reduce disparities in sexual health care and improve access to services for underserved populations.

## 2 Methods

### 2.1 Data collection

**Figure 1:**
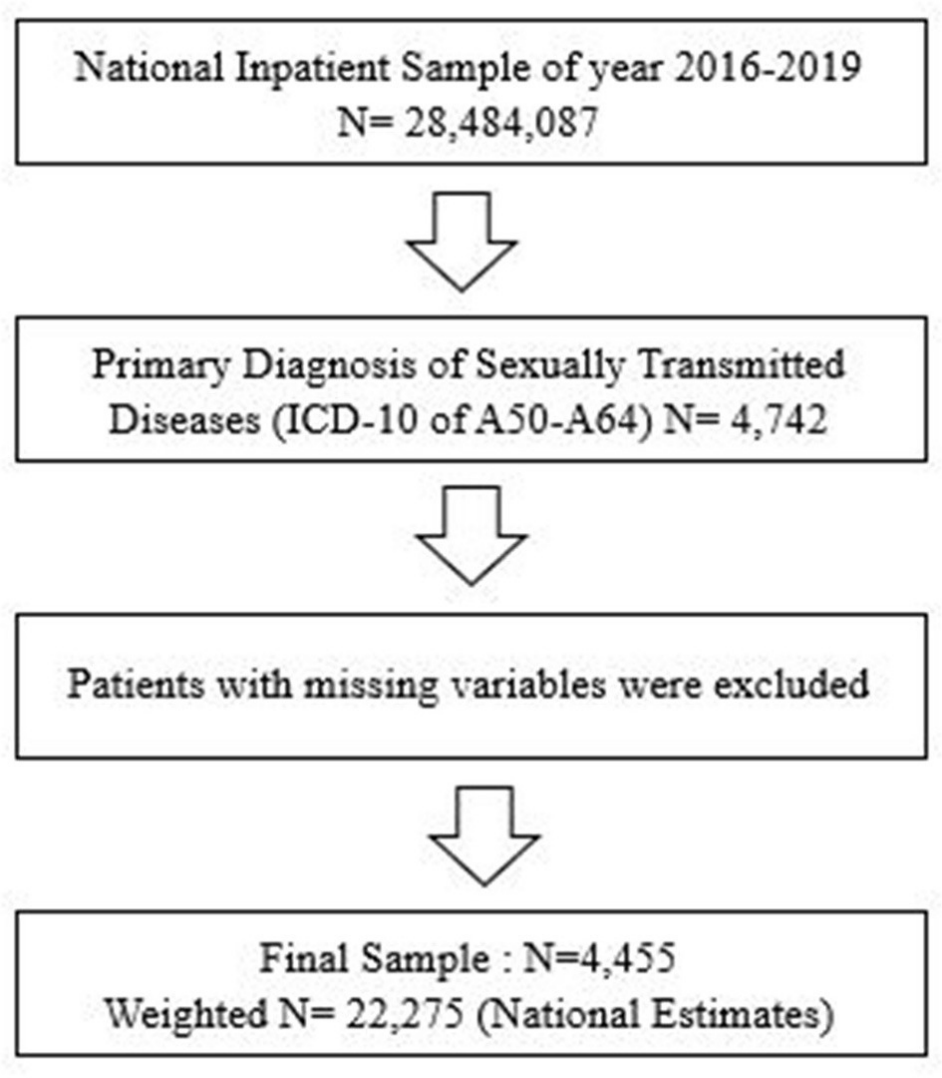
Flow chart of patient sample selection.

The latest 2016–2019 United States National Inpatient Sample (NIS) data were used to obtain a population-based estimate for patients with STDs. As shown in Figure 1, the study first identified the primary diagnosis of STDs (total *n* = 107,244) using the International Classification of Diseases, 10th Version (ICD-10) codes for STDs (A50–A64) among all 2016–2019 NIS samples (*N* = 28,484,087). After excluding patients with missing variables, this study obtained a final sample of patients with STDs (total *n* = 4,455, National Estimates = 22,275). Although the current study collected samples from the NIS data, they were independent of the NIS (Figure 1).

### 2.2 Variables

The primary outcomes of this study were to investigate the characteristics associated with higher odds of ER visits in patients with STDs and the association between ER visits and healthcare utilization as measured by hospital charges. Owing to the skewed distribution of hospital charges, the study conducted a natural log of this variable. Additionally, the data was adjusted for various patient- and hospital-level confounders. Patients' characteristics included age, race, annual median household income, primary payer (Medicare, Medicaid, Self-Pay/No Charge, Other, or Private insurance), and severity of illness. Hospital characteristics included bed size, ownership, location, teaching status, and the region where the patients were treated.

### 2.3 Statistical analysis

Sampling weights were used for all statistical analyses to represent patients with STDs nationwide. First, this study examined the characteristics of the final dataset. Patients' characteristics were presented as weighted frequency (percentage) or mean with standard deviation (SD). Second, this present study then investigated the temporal trends in patients, ER visits, and hospital charges among patients with STDs. Next, the data investigated how patients' characteristics were associated with an ER visit before inpatient care using multivariate logistic regression analysis. Finally, ER visits and other characteristics were analyzed to relate to hospital charges using a multivariate linear regression analysis. Additionally, the models using census division variables were used to determine more specific regional variances. Finally, subgroup analyses were performed according to race. All studies were conducted using the SAS statistical software (version 9.4; SAS Institute Inc., Cary, NC, USA). All statistical tests were two-sided, and statistical significance was set at *p* < 0.05.

## 3 Results

### 3.1 Patient characteristics

A total of 4,455 patients with STDs were identified in the 2016–2019 NIS data, with a weighted total of 22,275 nationally representative patients. Among these patients, 74% were associated with ER visits. The racial distribution of patients with STDs in the sample was relatively reflective of the general U.S. population. However, the sample included a higher proportion of Black patients (36.3%), and a lower proportion of other racial groups compared to national averages. In terms of demographics, a larger proportion of patients were female (57.9%), of low income, and from the Southern U.S. (44.3%). The detailed patient characteristics are shown in Table 1.

**Table 1:** General characteristics of the study sample.

**Variables**	**N**	**%**
***N***	4,455	
**Weighted N [national estimates]**	22,275	
**STD inpatients' emergency visit**		
Yes	16,490	74.0%
No	5,785	26.0%
**Race**		
White	9,215	41.4%
Black	8,095	36.3%
Hispanic	3,460	15.5%
Asian or Pacific Islander	460	2.1%
Native American	255	1.1%
Other	790	3.5%
**Age** ^*^		
**Sex**		
Male	9,370	42.1%
Female	12,905	57.9%
**Median household income**		
0–25th percentile	9,835	44.2%
26th to 50th percentile	5,540	24.9%
51st to 75th percentile	4,115	18.5%
76th to 100th percentile	2,785	12.5%
**Primary payer**		
Medicare	3,520	15.8%
Medicaid	8,875	39.8%
Private insurance	5,690	25.5%
Self-pay	3,370	15.1%
No charge	185	0.8%
Other	635	2.9%
**Severity of Illness**		
No/Minor comorbidity or complications	8,980	40.3%
Moderate comorbidity or complications	9,455	42.4%
Major comorbidity or complications	3,310	14.9%
Extreme comorbidity or complications	530	2.4%
**Bedsize of hospital**		
Small	3,790	17.0%
Medium	5,730	25.7%
Large	12,755	57.3%
**Ownership of hospital**		
Government, nonfederal	4,215	18.9%
Private, not-profit	15,575	69.9%
Private, invest-own	2,485	11.2%
**Location/teaching status of the hospital**		
Rural	1,125	5.1%
Urban nonteaching	3,435	15.4%
Urban teaching	17,715	79.5%
**Region of hospital**		
Northeast	3,560	16.0%
Midwest	4,235	19.0%
South	9,875	44.3%
West	4,605	20.7%

### 3.2 Temporal patterns of patients sample and hospital charges

Table 2 demonstrates the temporal trends for hospitalized STDs patients between 2016 and 2019. During this period, both the number of patients and the number of ER visits increased. Specifically, the total number of ER visits rose from 3,700 in 2016 to 4,795 in 2019, corresponding to an increase in the proportion of ER visits from 70.9% in 2016 to 76.4% in 2019. Additionally, average hospital charges increased over the study period, from $40,345 in 2016 to $47,432 in 2019.

**Table 2:** Temporal trend of the patient sample and hospital charges.

	**2016**	**2017**	**2018**	**2019**
***N***	1,043	1,077	1,079	1,256
**Weighted N [National Estimates]**	5,215	5,385	5,395	6,280
**Weighted N of Emergency Visit**	3,700	3,995	4,000	4,795
**% of Emergency visit**	70.9%	74.2%	74.1%	76.4%
**Average Hospital Charges**	40,345	43,832	43,062	47,432

### 3.3 Association between emergency visits and various patient characteristics

Table 3 presents the OR for ER visits derived from a multivariate logistic regression model. Several key findings emerge. Males were significantly less likely to visit the ER compared to females (OR = 0.746, 95% CI: 0.642–0.867). Regarding primary payers, Medicaid, and self-paid patients were more likely to visit the ER compared to those with private insurance. Specifically, the odds of an ER visit were 1.22 times higher for Medicaid patients (95% CI: 1.021–1.464) and 2.57 times higher for self-pay patients (95% CI: 1.993–3.323). These findings point to significant disparities in access to care, with individuals on Medicaid or with no insurance being more likely to rely on emergency services. In terms of region, patients from the South (OR = 1.271, 95% CI: 1.056–1.530) and West (OR = 1.339, 95% CI: 1.071–1.675) were more likely to visit the ER than those from the Midwest.

**Table 3:** Results of survey logistic regression: odds of an emergency visit by different patient characteristics.

**Variables**	**Odds ratio**	**95% CLs**	
**Race**			
White	Ref.		
Black	1.096	0.926	1.297
Hispanic	0.972	0.790	1.197
Asian or Pacific Islander	1.364	0.805	2.312
Native American	0.860	0.452	1.638
Other	0.817	0.560	1.192
**Age**	1.000	0.995	1.005
**Sex**			
Male	0.746	0.642	0.867
Female	Ref.		
**Median household income**			
0–25th percentile	Ref.		
26th to 50th percentile	0.962	0.808	1.146
51st to 75th percentile	0.975	0.799	1.191
76th to 100th percentile	1.110	0.872	1.413
**Primary payer**			
Medicare	0.885	0.685	1.144
Medicaid	1.223	1.021	1.464
Private insurance	Ref.		
Self-pay	2.573	1.993	3.323
No charge	1.562	0.711	3.431
Other	0.776	0.525	1.147
**Severity of Illness**			
No/Minor comorbidity or complications	Ref.		
Moderate comorbidity or complications	1.006	0.862	1.174
Major comorbidity or complications	1.246	0.991	1.568
Extreme comorbidity or complications	1.096	0.678	1.773
**Bedsize of hospital**			
Small	Ref.		
Medium	0.856	0.688	1.065
Large	0.901	0.740	1.096
**Ownership of hospital**			
Government, nonfederal	Ref.		
Private, not-profit	1.262	1.052	1.515
Private, invest-own	1.166	0.893	1.523
**Location/teaching status of the hospital**			
Rural	Ref.		
Urban nonteaching	1.326	0.931	1.886
Urban teaching	1.097	0.806	1.494
**Region of hospital**			
Northeast	2.225	1.721	2.877
Midwest	Ref.		
South	1.271	1.056	1.530
West	1.339	1.071	1.675
**Year**	1.096	1.031	1.165

### 3.4 Association of emergency visits and other characteristics with hospital charges

Table 4 shows the results of a linear regression model examining the association of ER visits and other characteristics with hospital charges. The key findings include that ER visits were significantly associated with higher hospital charges. The estimated increase in hospital charges for patients who visited the ER was 13.5% (*p* < 0.0001). In terms of race, Hispanic patients incurred significantly higher hospital charges compared to White patients (β = 0.128, *p* < 0.0001). Concerning primary payers, patients on Medicaid had significantly higher hospital charges compared to those with private insurance (β = 0.067, *p* = 0.028). Regionally, compared to the Midwest, hospital charges were significantly higher in the South (β = 0.140, *p* < 0.0001), West (β = 0.528, *p* < 0.0001), and Northeast (β = 0.252, *p* < 0.0001).

**Table 4:** Results of the linear survey regression: association with hospital charges.

**Variables**	**Hospital Charges**	
	**Est**.	**P**
**Emergency visit**		
Yes	0.135	< 0.0001
No	Ref.	
**Race**		
White	Ref.	
Black	0.047	0.084
Hispanic	0.128	< 0.0001
Asian or Pacific Islander	0.014	0.870
Native American	−0.195	0.049
Other	0.210	0.002
**Age**	0.004	< 0.0001
**Sex**		
Male	0.181	< 0.0001
Female	Ref.	
**Median household income**		
0–25th percentile	Ref.	
26th to 50th percentile	0.010	0.719
51st to 75th percentile	0.011	0.744
76th to 100th percentile	0.021	0.599
**Primary payer**		
Medicare	0.086	0.065
Medicaid	0.067	0.028
Private insurance	Ref.	
Self-pay	0.055	0.118
No charge	0.107	0.315
Other	0.185	0.022
**Severity of Illness**		
No/Minor comorbidity or complications	Ref.	
Moderate comorbidity or complications	0.240	< 0.0001
Major comorbidity or complications	0.594	< 0.0001
Extreme comorbidity or complications	1.516	< 0.0001
**Bedsize of hospital**		
Small	Ref.	
Medium	0.014	0.688
Large	0.099	0.001
**Ownership of hospital**		
Government, nonfederal	Ref.	
Private, not-profit	0.079	0.010
Private, invest-own	0.520	< 0.0001
**Location/teaching status of the hospital**		
Rural	Ref.	
Urban nonteaching	0.242	< 0.0001
Urban teaching	0.375	< 0.0001
**Region of hospital**		
Northeast	0.252	< 0.0001
Midwest	Ref.	
South	0.140	< 0.0001
West	0.528	< 0.0001
**Year**	0.060	< 0.0001

### 3.5 Models with specific region variable and sub-group analysis by race

Table 5 presents the results from a model that replaced the region variable with the Census Division, as well as a subgroup analysis by race. Key findings include those hospitals in the New England region had significantly lower charges compared to the South Atlantic (β = −0.209, *p* = 0.002). Conversely, hospitals in the Middle Atlantic (β = 0.252, *p* < 0.0001), West South Central (β = 0.075, *p* = 0.041), Mountain (β = 0.163, *p* = 0.003), and Pacific (β = 0.513, *p* < 0.0001) regions had higher hospital charges. Regarding the subgroup analysis by race, ER visits were associated with higher hospital charges, particularly among White (β = 0.114, *p* = 0.011) and Black (β = 0.177, *p* = 0.001) populations. Interestingly, hospital charges were not significantly higher for Hispanics (β = 0.102, *p* = 0.183), Asian or Pacific Islanders (β = 0.056, *p* = 0.757), Native American (β = −0.158, *p* = 0.691), or Other racial groups.

**Table 5:** Results of the linear survey regression: replace region variable by census division and sub-group analysis by race.

**Variables**	**Hospital charges**	
	**Est**.	***P***
**Census division of hospital**		
New England	−0.209	0.002
Middle Atlantic	0.252	< 0.0001
East North Central	−0.077	0.034
West North Central	−0.198	< 0.0001
South Atlantic	Ref.	
East South Central	0.038	0.437
West South Central	0.075	0.041
Mountain	0.163	0.003
Pacific	0.513	< 0.0001
White	0.114	0.011
Black	0.177	0.001
Hispanic	0.102	0.183
Asian or Pacific Islander	0.056	0.757
Native American	−0.158	0.691
Other	−0.014	0.925

All other Variables were adjusted.

## 4 Discussion

The current study found differences in hospital costs for various patient factors that indicate health disparities affecting minorities and the South. Such differences can significantly impact patients with limited finances and are essential for identifying areas of concern to target financial and preventative support. We found higher STDs rates in females, low-income individuals, and the South- already of concern for historically high STDs rates and limited financial means (1, 16). Further, males with STDs were more likely to have ER visits, and ER visits were significantly associated with higher overall hospital charges. This is a novel finding that coincides with increasing gonorrhea rates in men (1), showing that this population is of growing interest. Because STIs can be asymptomatic (2), catching them in men presenting to the ER before progressing to an STDs and acquiring potential at-risk partners can limit further costs as inpatients and promote preventive efforts. Medicaid and self-payers had some of the highest risks for ER visits, which is concerning because we found that ER visits were associated with higher hospital charges.

Certain regions, such as the South and Deep South, are known to have higher STDs rates (1, 12, 13). This present study found similarly high STDs trends in the south, although it had one of the lower rates of ER visits and hospital charges compared to the Northeast and West. The Northeast and West had higher ER rates, possibly contributing to their higher hospital charges. Both areas also have higher medical consumer price indices, the two highest in the nation (17–20), which could be reflected in their higher costs. The decreased healthcare utilization in the south, presented by lower ER rates and hospital charges, could be due to two primary factors. First, the South has long struggled with reduced access to care, characterized by higher stigma, provider shortages, and low health literacy (13). Second, the South had the highest poverty rates (16) and lowest median incomes (16) in 2019, further limiting access to necessary healthcare services. Despite facing higher STDs rates, patients in the South may not receive proper care. This situation is exacerbated by the lack of federal financial assistance for diseases such as HIV (12) and limited Medicaid expansion in the South. These factors likely contribute to the higher ER rates for Medicaid patients, which highlight a critical area of concern. The low healthcare costs observed in the South could indicate not improved efficiency, but rather a lack of access to appropriate care due to these systemic barriers. It is essential to acknowledge potential confounding factors in this study, particularly the varying state policies on healthcare and Medicaid expansion, which could significantly influence the results. For example, states with expanded Medicaid coverage may see higher rates of healthcare access, potentially leading to more frequent but less costly care in non-emergency settings. These variations in healthcare policies could be critical confounders that impact both ER visit rates and hospital charges, which should be considered in future research and policymaking aimed at reducing health disparities.

Looking at how hospital charges differ based on race and census region, our model shows disparities across more targeted areas. The Middle Atlantic and Pacific regions had significantly higher hospital charges, while the West South Central, and the Mountain regions had non-significant but higher hospital charges. Conversely, the West North Central region was the only region with significantly lower hospital charges than the South Atlantic. Focusing on the significant differences, the Pacific region had the highest hospital charges, which coincides with the high medical consumer price index (CPI; 18), high ER rates, and high hospital charges in the West. The Middle Atlantic in the Northeast followed a similar pattern. The West North Central in the Midwest was the only significant decrease. The Midwest was the reference for hospital charges and ER visits and the second-lowest for income and medical CPI (16, 19). Thus, similar to the South, this region's lower costs may indicate decreased utilization due to decreased access to care.

Racial disparities were not as apparent, with the only significant association being with increased hospital charges in Hispanic patients. Hispanic patients had significant, albeit slight, increased hospital charges, as shown in Table 4. Past data have shown that Hispanic patients are challenged by increasing HIV (21), syphilis, and gonorrhea cases (1). Their increasing disease rates and high hospital charges, when compounded with their second-lowest median incomes (16), signal an increased financial barrier to care. Previous research has indicated that to improve outcomes in this population, programs must be individualized according to their cultural differences, and Hispanic patients should be better represented in research (21).

Other factors influenced hospital charges, including hospital ownership, location/teaching status, and year. Private investor-owned hospitals have significantly higher costs. The year was also associated with higher payments, and we noted that the charges increased by $7,000 between 2016 and 2019. The current study found that rural hospitals had significantly lower costs than urban teaching and non-teaching hospitals. Although rural areas often experience higher STDs rates, access to care in these areas is limited (1, 13, 14). The lower hospital charges in rural areas may reflect the fact that patients in these areas are less likely to seek care due to barriers such as stigma, distance to facilities, and lower health literacy. Policy interventions should focus on increasing federal funding for sexual health services and improving infrastructure in rural areas to enhance access to care. Additionally, rural areas could benefit from mobile health clinics and telemedicine services to reduce barriers to care. These findings underscore the need for increased federal funding and sexual health education in rural areas, which could improve access to STDs care.

This study can have important implications for existing STDs prevention programs, including those focusing on HIV pre-exposure prophylaxis (PrEP) and improving healthcare access in underserved regions. Given the significant disparities in healthcare utilization and costs identified in the South and rural areas, policy interventions should prioritize improving access to PrEP, especially for at-risk populations in these regions. Expanding Medicaid coverage in states that have not yet expanded, increasing funding for local STDs clinics, and improving awareness of available preventive measures like PrEP could help reduce reliance on emergency services and prevent the progression of STDs to more severe stages. Additionally, targeted outreach programs should focus on educating populations that face barriers to care, including low-income individuals, those with limited access to healthcare, and racial minorities, to promote prevention and early treatment of STDs. Programs that address stigma, improve provider-patient communication, and increase awareness of testing and treatment options are crucial in promoting better sexual health outcomes.

This study characterized the differences in hospital charges and ER usage by race, region, and other patient and hospital factors to identify at-risk populations with more significant barriers to care. However, our study had some limitations. First, the ICD-10 codes for STDs used by the NIS limited patient selection. This approach may have excluded patients with unrecorded or miscoded diagnoses, which could result in an underrepresentation of individuals who have STDs but were not accurately coded. Such exclusions or misclassifications could introduce bias, potentially affecting the generalizability of the findings by failing to capture all patients who might be at risk for increased healthcare costs or ER visits. Second, clinical information or disease severity, connected to increased cost, was not included in the dataset, restricting real-life interpretation and weakening the study results. Another limitation involves the exclusion of patients with missing variables. The exclusion of these patients may have impacted the representativeness of the final sample, as it could have led to the loss of certain subgroups of interest, particularly those with missing data for variables such as insurance status or comorbidities. This could introduce bias if the missing data were not missing at random, which would limit the ability to generalize the findings to the entire population of STDs patients. In addition, the annual medical services CPI was not available for all four census regions, only for the Northeast and West. Instead, the CPI for December 2019 was used. This information was available for all four areas during the last month of the study period. Finally, the dataset did not include information on inpatient or outpatient care or the perspectives of patients and physicians on STDs. Despite these limitations, our study was well-sampled over multiple study periods and is generalizable to most STDs patients in the US. Our study has demonstrated significant healthcare disparities surrounding financial barriers to STDs care, which call for more outstanding research and resource allocation.

## 5 Conclusions

The present study demonstrated the importance of understanding patient factors and involving community input in tailoring treatment, prevention, and monitoring programs for STDs. Doing so can lower hospital charges by promoting STDs clinics, reducing stigma, and providing better sexual health education. Specific policy recommendations include increasing federal funding for STDs clinics, especially in underserved regions like the South and rural areas, enhancing access to Medicaid and other insurance programs, and focusing on preventive sexual health education to reduce the need for ER visits. These efforts may prevent ER visits, thus decreasing overall payments, and could prevent infections from progressing to inpatient diseases. Unfortunately, Hispanics, rural locations, and southern regions are some of the patient populations we have identified as at risk for increased barriers to care. For example, Hispanic patients had some of the highest costs, potentially reflecting their increased healthcare utilization due to growing STDs rates, but also some of the lowest median incomes. Conversely, the southern and rural areas had lower hospital charges, but this may be a sign of decreased healthcare access and utilization due to surrounding stigmas, distance to facilities, and lower health literacy. Policy interventions should specifically target these regions and populations with additional resources, education, and healthcare services to mitigate these disparities. The disparities in populations we identified are multifactorial and require more research and resources to overcome.

## Data Availability

The original contributions presented in the study are included in the article/supplementary material, further inquiries can be directed to the corresponding author.
